# Metabolic Plasticity of Stem Cells and Macrophages in Cancer

**DOI:** 10.3389/fimmu.2017.00939

**Published:** 2017-08-09

**Authors:** Jelena Krstic, Drenka Trivanovic, Aleksandra Jaukovic, Juan F. Santibanez, Diana Bugarski

**Affiliations:** ^1^Laboratory for Experimental Hematology and Stem Cells, Institute for Medical Research, University of Belgrade, Belgrade, Serbia; ^2^Institute of Cell Biology, Histology and Embryology, Medical University Graz, Graz, Austria

**Keywords:** metabolism, cancer stem cells, mesenchymal stem cells, macrophages, tumor microenvironment, cancer, plasticity

## Abstract

In addition to providing essential molecules for the overall function of cells, metabolism plays an important role in cell fate and can be affected by microenvironmental stimuli as well as cellular interactions. As a specific niche, tumor microenvironment (TME), consisting of different cell types including stromal/stem cells and immune cells, is characterized by distinct metabolic properties. This review will be focused on the metabolic plasticity of mesenchymal stromal/stem cells (MSC) and macrophages in TME, as well as on how the metabolic state of cancer stem cells (CSC), as key drivers of oncogenesis, affects their generation and persistence. Namely, heterogenic metabolic phenotypes of these cell populations, which include various levels of dependence on glycolysis or oxidative phosphorylation are closely linked to their complex roles in cancer progression. Besides well-known extrinsic factors, such as cytokines and growth factors, the differentiation and activation states of CSC, MSC, and macrophages are coordinated by metabolic reprogramming in TME. The significance of mutual metabolic interaction between tumor stroma and cancer cells in the immune evasion and persistence of CSC is currently under investigation.

## Introduction

Cells constituting tumor microenvironment (TME), such as immune cells, endothelial cells, fibroblasts, and mesenchymal stromal/stem cells (MSC), communicate with cancer cells mutually influencing properties of each cell type and overall outcome of tumor growth (1). Predominantly engaged in turnover of vital biomolecules, energy metabolism is an important regulator of cell fate and functions, while it can be modified through the crosstalk with microenvironmental cues. The lever between two general metabolic processes, anabolism and catabolism, can be shifted to respond to cells’ needs (2). When it comes to stem cells, general opinion is that the pluripotent, quiescent state of these cells drives or is driven by a glycolytic profile, while in the state of differentiation stem cells turn to oxidative phosphorylation (OXPHOS) for energy production (2, 3). In TME, not only cancer cells are able to reprogram their metabolism according to their needs but also they can reprogram the metabolism of surrounding cells to respond to their demands and fuel tumor growth (4, 5). Although the specificity of cancer metabolism is partly recognized, the metabolic properties and mutual interactions of each cell type within TME need to be revealed in order to understand the complex metabolic crosstalk within. This review will focus on the metabolic properties in three compartments of TME: cancer stem cells (CSC), MSC, and macrophages as major component of leukocytic infiltrate in tumors.

## Intrinsic Heterogeneity of Cancer Cell Metabolism—Place to be for CSC

The ability to acquire necessary nutrients from an often poor nutrient content within the environment and to produce molecules required for its own expansion is a hallmark of cancer metabolism (5). Oncogenic mutations lead to metabolic reprogramming of cancer cells that support carcinogenesis. The alterations in intracellular and extracellular metabolites have profound effects on gene expression, cellular differentiation, and the TME of cancer cells. Cancer-driven metabolic features include deregulated uptake of glucose and amino acids, use of glycolysis/tricarboxylic acid (TCA) cycle intermediates for NADPH production, genesis of oncometabolites, and metabolic interactions with TME (5, 6). Although glycolytic, many cancer cells produce ATP in mitochondria, since, besides pyruvate, fatty acids and amino acids can supply substrates to the TCA cycle. Therefore, cancer cells have an opportunity to adapt their metabolic pathways to the microenvironment. Cancer cells adjacent to blood vessels use nutrients and oxygen for anabolism, while cancer cells away from vessels have to include other metabolic pathways, such as oxidation of fatty acids or recycling of molecules *via* autophagy (5).

**Figure 1:**
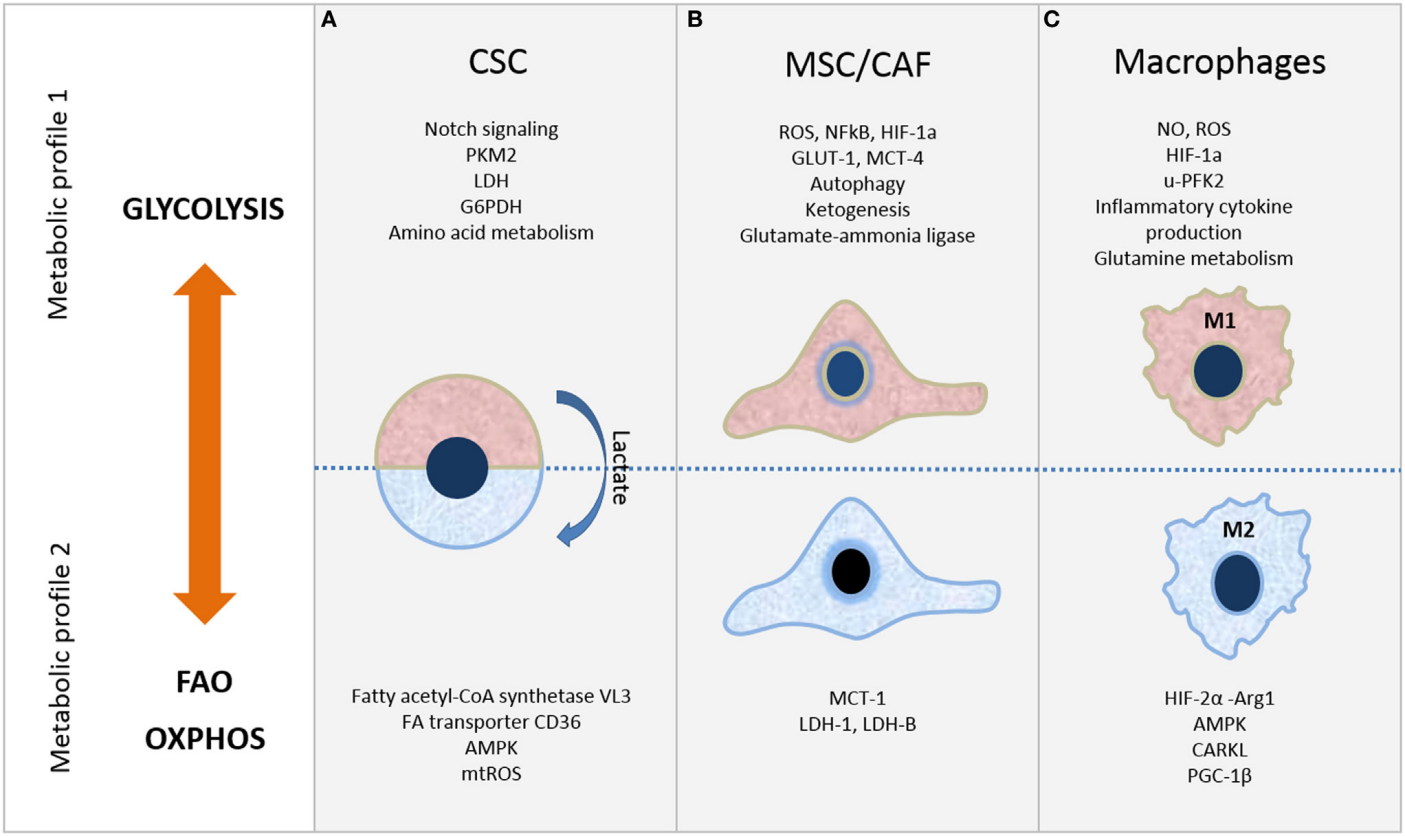
Metabolic plasticity of cells in tumor microenvironment. Selected metabolic features of **(A)** cancer stem cells (CSC), **(B)** mesenchymal stromal/stem cells (MSC)/cancer-associated fibroblasts (CAF), and **(C)** macrophages. Refer to the text for further details.

Cancer persistence is associated with existence of CSC, which are recognized as cells with accumulated mutations, ability to differentiate/transdifferentiate (7) and self-renew (8). Even though metabolic features of CSC are not yet revealed, it is possible to speculate that, in comparison to normal stem cells, CSC with the mutated genome have greater opportunity to adapt to microenvironmental circumstances by modulating their energy production pathways (9). It has been accepted that CSC have glycolytic metabolic phenotype, while more differentiated cells rely on OXPHOS. This notion is partly associated with the switch from OXPHOS to glycolysis during reprogramming and achieving of pluripotency initiated by transcription factors, Sox2, Oct4, Klf4, or Myc in iPS cells (10). However, CSC with OXPHOS profile were shown to be resistant to inhibition of glycolysis and more independent from microenvironment nutrient level. Importantly, CSC can also rely on mitochondrial fatty acid oxidation (FAO) (11) for ATP and NADPH generation (12, 13). Thus, CSC with OXPHOS profile may acquire a selective advantage in specific TME, as they use limited nutrients more efficiently. Lactate, excreted by more differentiated cancer cells that are dependent on glycolysis, may in return serve as fuel for OXPHOS in CSC that depend on mitochondrial metabolism, consequently establishing a metabolic symbiosis system (12, 14) (Figure 1A).

Depending on the cancer type, CSC show distinct metabolic profiles that can be glycolysis or OXPHOS dependent (Figure 1A). In either case, mitochondrial function is critical and exhibits crucial role in CSC metabolism. The changeable metabolism of CSC population in various cancer types will be discussed next.

There are inconsistent results regarding metabolic feature of CSC within lung cancer. As for CSC within small-cell lung cancer cell line H446, OXPHOS metabolic profile, lower oxygen consumption rate, and acidification compared to non-stem-like cells were shown (15). Yet, another study reported that side population in lung cancer cells which export Hoechst 33342 and chemotherapeutics has high glycolytic activity (16).

Similarly, uneven results can be observed for breast cancer CSC. Glycolytic profile of CSC and non-stem cancer cells within breast was confirmed (17). Enhanced Notch signaling was shown to support self-renewal of breast CSC with high glycolytic activity associated with progressive hormone-independent growth *in vivo*. Hormonal therapy induces OXPHOS metabolic editing of luminal breast cancers, establishing self-renewal of dormant CD133^high^/estrogen receptor (ER)^low^ cells (18). In contrast, cultivation of breast cancer cells in non-adherent conditions fosters shift from OXPHOS toward glycolysis, increasing activity of anaerobic glycolysis enzymes, such as pyruvate kinase M2 isoform, lactate dehydrogenase, and glucose 6-phopshate dehydrogenase in breast CSC (19).

In pancreatic ductal adenocarcinoma (PDA), CSC also rely on glycolysis, as glycolysis inhibitor, 3-bromopyruvate, attenuates self-renewal potential, aldehyde dehydrogenase 1 (ALDH1) activity, and reverts gemcitabine resistance (20). Stimulation of glucose transporter 1 (GLUT-1) expression has been related to the maintenance of CSC population in pancreas, ovarium, and brain in *in vivo* animal models, thus indicating importance of glucose metabolism for these CSC (21). CSC within PDA can also utilize non-canonical glutamine pathway. Glutamine deprivation caused attenuated self-renewal ability, decreased expression of stemness genes, and induced apoptosis in pancreatic CSC (22).

On the other hand, ovarian CSC are not limited to aerobic glycolysis but are amino acid metabolism dependent, especially for serine, aspartate, glutamate, and glutamine (23). Particularly, lipid metabolism is involved in CSC maintenance. It has been shown that the fatty acetyl-CoA synthetase VL3 (ACSVL3) is involved in glioblastoma genesis, while neurospheres of glioblastoma CSC have high level of ACSVL3 expression, associated with expression of several stemness markers, such as CD133, ALDH, Musashi-1, and Sox-2 (24). In accordance, fatty acid synthase (FASN), key lipogenic enzyme, can attenuate stemness in glioma cells, while their differentiation abolishes FASN expression (25). Also, fatty acids derived through lipolysis in gonadal fat can fuel FAO in leukemic stem cells (LSCs) which express high level of fatty acid transporter CD36, contributing to high chemoresistance of LSC (26). Hypoglycemic condition in the bone marrow (BM) favors survival of LSC which are more dependent on AMPK-suppression of oxidative stress than LSC in spleen, thus indicating tissue context-dependent metabolic activity of LSC (27).

## Metabolic Reprogramming of MSC Fuels Cancer Growth

Among the stromal cells of TME, MSC have recently drawn great attention. These adult stem cells play important role in tissue homeostasis and repair due to their self-renewal and multilineage differentiation capacity. Aside from the well-known feature of MSC to migrate and home tumors, there are conflicting reports regarding whether they promote or suppress tumor growth (28). Even less is known about the role of MSC metabolism in carcinogenesis. However, the capacity of resident or recruited MSC to differentiate into cancer-associated fibroblasts (CAF) (29) which affect cancer cell proliferation and invasiveness through secretion of growth factors, cytokines, and various metabolites has been well recognized. Indeed, mutual interactions between cancer cells and CAF have been found as the most important metabolic crosstalk in TME where metabolic asymmetry between these cell compartments critically drives tumor growth (30).

In particular, in many types of human tumors, including breast, prostate, head and neck cancers, and lymphomas, cancer cells metabolically reprogram CAF toward glycolytic phenotype, increasing their glucose uptake and lactate secretion (31) (Figure 1B). This reprogramming of CAF toward catabolic behavior is mediated by increased expression of GLUT-1 and monocarboxylate transporter-4 (MCT-4) enabling lactate efflux. In turn, cancer cells upload lactate through the MCT-1 and consume it for ATP synthesis through the TCA and OXPHOS undergoing reverse Warburg metabolism or metabolic coupling (32). Moreover, cancer cells use these molecules in anabolic pathways to provide biomass for cell proliferation (4). Similar metabolic coupling was evidenced between glycolytic adipose tissue-derived MSC which secreted lactate and expressed higher levels of MCT-4 and osteosarcoma cells which consumed lactate for ATP production and OXPHOS by increased expression of MCT-1 (33).

Other findings demonstrated that catabolic CAF also produce other metabolites, such as ketone bodies and glutamine which can fuel mitochondria of cancer cells and drive their OXPHOS and growth. Overexpression of ketogenic enzymes (e.g., mitochondrial 3-hydroxy-3-methylglutaryl CoA synthase) was found in CAF, while enzymes associated with ketone reutilization (e.g., ACAT1) were shown to be upregulated in cancer cells (34, 35). Moreover, it has been shown that autophagy in CAF can be induced by ketogenesis-derived ketone bodies (36), implying the reciprocal relationship between these processes. The role of CAF in glutamine metabolism has been recently studied showing increased expression of glutamate-ammonia ligase, key enzyme of glutamine synthesis, in CAF upon culture with cancer cells (37). Furthermore, CAF-derived glutamine was found to increase mitochondrial biogenesis and favor OXPHOS in cancer cells by decreasing their autophagy (4, 38). Mesenchymal stem-like cells (MSLC), isolated from malignant pleural effusion or ascites of lung, breast, and ovarian cancer patients, were also shown to transfer energy and biomass by glutamine to cancer cells (39). Moreover, it was proposed that glutamine and ammonium form a vicious cycle between MSLC and cancer cells. Namely, glutamine released by MSLC was used by cancer cells which further catabolized it to ammonia giving ammonium upon extracellular secretion. Interestingly, this ammonium was absorbed by MSLC promoting their growth.

Intensive investigations of mechanisms underlying metabolic coupling in cancer revealed critical role of nuclear factor kappa-B and hypoxia-inducible factor-1α (HIF-1α) transcription factors activation in CAF by pseudo-hypoxic state which has been generated *via* cancer cell-driven inflammation and oxidative stress (40). High reactive oxygen species (ROS) levels produced by cancer cells were found to induce loss of Caveolin-1 in CAF leading to increased autophagy and ketone bodies generation along with enhanced glycolysis mediated through HIF-1α stabilization (41). Moreover, enhanced OXPHOS activity, increased proliferation and invasiveness of breast cancer cells were evidenced upon nanotubes-mediated transfer of BM-MSC-derived mitochondria to cancer cells (42).

However, mutual metabolic cooperation between stromal and cancer cells is very dynamic and even more sophisticated, as opposite metabolic interplay between them was reported. In colorectal carcinoma, opposite behavior of CAF was suggested, as increased expression of lactate dehydrogenase (LDH)-5 and MCT-4 related to glycolytic metabolism was shown in cancer cells, while high levels of LDH-1 and MCT-1 indicative of OXPHOS was found in CAF (Figure 1B) (43). Similarly, BM-MSC and MSC-derived CAF were shown to upload lactate secreted by breast cancer cells *via* MCT1 and to convert it *via* LDH-B into pyruvate (44) and glycolytic metabolism of melanoma cells (45). More recently, CAF were demonstrated to inhibit OXPHOS while inducing glycolysis in prostate cancer cells through delivery of extracellular vesicles (EV), which were shown to transfer TCA cycle metabolites, amino acids and lipids (46). Novel findings showing that EV derived from serum-deprived BM-MSC contain glutamic and lactic acid, as well as microRNAs which regulate metabolism-associated genes, indicate that MSC can also affect osteosarcoma cell metabolism and growth through cargo content of their EV (47, 48).

## Metabolic Reprogramming Drives Macrophages Fate in TME

Macrophages are versatile innate immune cells that play crucial roles in normal tissue homeostasis as well as in several pathological conditions (49, 50). Tumor-associated macrophages (TAM) are present in large proportion in TME and play a key role in tumorigenesis (51). Under different microenvironmental signals macrophages undergo different states of activation: the classical activation or M1 (“killing phenotype”) and the alternative activation or M2 (“healing phenotype”). Briefly, the M1 state is proinflammatory and it is induced by endotoxin, interferon-γ, and/or interleukin-1α (IL-1α), while M2 state is anti-inflammatory and it is involved in the resolution of inflammation, induced by IL-4, IL-10, IL-13, transforming growth factor (TGF)-β, and glucocorticoids (49, 52). Most TAM have the M2 phenotype due to the signals in the TME, such as IL-4 and TGF-β (51). Also, TAM can increase the number, drug resistance, and tumorigenicity of CSC, while CSC are able to induce the M2 phenotype (51).

Importantly, macrophages require changes in the intracellular metabolism for proper polarization. M1 macrophages express high glycolysis rate and release lactate in parallel to the reduction in oxygen consumption, allowing their survival in low oxygen microenvironment found in cancer and chronic inflammatory sites. On the other hand, M2 macrophages preferably exploit OXPHOS and FAO (53–55) as shown in Figure 1C.

There is an inverse relation between regulators of M1 and M2 macrophages. For instance, in classical activation of peritoneal macrophages a strong expression of u-phosphofructokinase 2 (u-PFK2) isoform concomitant with increased levels of Fru-2,6-P2 occurs, and u-PFK2 seems to promote glycolysis (56, 57). On the other hand, the sedoheptulose kinase CARKL, that limits the pentose pathway, is upregulated in M2 and its expression is strongly reduced in M1 (58). Furthermore, in M1 macrophages the Krebs cycle is stopped at two different points. First, after citrate step; citrate accumulation appears to be essential for the synthesis of proinflammatory regulators including ROS, nitric oxide (NO), and prostaglandins among others (57). Cytosolic citrate participates in phospholipids synthesis *via* its conversion, by the citrate lyase, into acetyl-CoA, thus providing substrates for arachidonic acid, which is critical for the production of prostaglandins. Similarly, oxaloacetate is produced and used for NADPH generation through its conversion into malate by the cytosolic malic enzyme, which participates in both NO and ROS generation. In addition, NADPH is also produced by pentose-phosphate pathway that is strongly activated during classical activation of macrophages (57, 59). Second, after succinate step; accumulation of succinate plays a critical role for IL-1α induction by lipopolysaccharide (LPS), since it leads to HIF-1α activation by inhibition of prolyl hydroxylases. Activated HIF-1α further transcriptionall activates IL-1α promoter thus triggering IL-1α expression (60). Conversely, HIF-2α activation is mainly observed in mouse M2 macrophages to induce arginase-1 (Arg1) expression and inhibit NO production (61) (Figure 1C). Also, M1-polarized macrophages present active glutamine metabolism. Glutamine is, *via* glutaminolysis, metabolized to Krebs cycle, stimulating α-ketoglutarate and succinate production and HIF1α activation, which is critical for IL-1α expression (62).

Meanwhile, M2 macrophages have an intact Krebs cycle having a primacy over glycolysis. The high active oxidative glucose metabolism (OGM) in IL-4-induced M2 provides the required energy for its contribution to tissue repair and regeneration (57, 63). For example, the related expression of Arg1 with the metabolism of arginine to proline is implicated in collagen production, which is required for tissue repair during resolution of inflammation (64). Both IL-4 and IL-13 are inducing OGM through inhibition of mechanistic target of rapamycin (mTOR). This mTOR inhibition causes reduced expression of HIF-1α which is further related to decreased expression of glycolytic and inflammatory response genes (65). Also, M2 polarization is highly dependent on FAO, which is critical for providing carbons to Krebs cycle. Triglycerides, the main source of fatty acids, are uptaken through CD36 and then subjected to lysosomal acid lipase hydrolysis, which is induced by IL-4 (57, 66). FAO induction is dependent on both STAT6 and peroxisome proliferator-activated receptor-γ-coactivator-1β (PGC-1β). Interestingly, PGC-1β is vital for IL-4-induced FAO, since RNAi knockdown or constitutive expression of PGC-1β either inhibit or increase FAO after IL-4 treatment, respectively (57, 67). Furthermore, lipid metabolism contributes to the regulation of membrane fluidity in macrophages phagocytosis process (68).

Another important feature of M2-polarized macrophages is the high AMPK activity, which plays a role as a key sensor of energy status for OXPHOS and FAO (64) (Figure 1). Furthermore, glutamine catabolism is also active in M2 polarization, but in this case relative to uridine diphosphate N-acetylglucosamine (UDP-GlcNAc) production, which is used for the N-glycosylation of mannose-binding lectin, a key mediator of M2 functions (57, 59).

## Conclusion

The complexity of TME arising from the variety of cell types present within is additionally enlarged by the heterogeneity of their metabolic state which can be modulated in such manner to sustain or stimulate tumor growth. As discussed in this review, metabolic signature can define whether stem cells are in their quiescent or proliferative state, it can define whether cells are being used as “feeder” cells by the tumor, or, in case of macrophages, whether they act as pro- or anti-inflammatory cells (summarized in Figure 1). Understanding the metabolic properties of the CSC entity that is associated with cancer occurrence and survival may help to define their elusive phenotype. Comprehension of the metabolic state of each cell population, particularly due to cells’ interaction within tumor stroma, can also open possibilities to develop new therapeutic targets by specific metabolic reprogramming of each of the three aforementioned cell types. More efficient antitumor therapies will have to consider simultaneous targeting of all metabolic compartments in TME.

## Author Contributions

JK, DT, AJ, and JFS performed literature searches and cowrote the review. These four authors contributed equally. JK conceived the minireview topic. DB cowrote and gave final approval of the review.

## Conflict of Interest Statement

The authors declare that the research was conducted in the absence of any commercial or financial relationships that could be construed as a potential conflict of interest.

## References

[1] Ye J Wu D Wu P Chen Z Huang J The cancer stem cell niche: cross talk between cancer stem cells and their microenvironment. Tumour Biol (2014) 35(5):3945–51. 10.1007/s13277-013-1561-x 24420150

[2] Chandel NS Jasper H Ho TT Passegué E. Metabolic regulation of stem cell function in tissue homeostasis and organismal ageing. Nat Cell Biol (2016) 18(8):823–32. 10.1038/ncb3385 27428307

[3] Chen CT Hsu SH Wei YH. Mitochondrial bioenergetic function and metabolic plasticity in stem cell differentiation and cellular reprogramming. Biochim Biophys Acta (2012) 1820(5):571–6. 10.1016/j.bbagen.2011.09.013 21983491

[4] Martinez-Outschoorn UE Lisanti MP Sotgia F Catabolic cancer associated fibroblasts transfer energy and biomass to anabolic cancer cells, fueling tumor growth. Semin Cancer Biol (2014) 25:47–60. 10.1016/j.semcancer.2014.01.005 24486645

[5] DeBerardinis RJ Chandel NS. Fundamentals of cancer metabolism. Sci Adv (2016) 2(5):e1600200. 10.1126/sciadv.1600200 27386546 PMC4928883

[6] Pavlova NN Thompson CB. The emerging hallmarks of cancer metabolism. Cell Metab (2016) 23(1):27–47. 10.1016/j.cmet.2015.12.006 26771115 PMC4715268

[7] Huang Z Wu T Liu AY Ouyang G Differentiation and transdifferentiation potentials of cancer stem cells. Oncotarget (2015) 6(37):39550–63. 10.18632/oncotarget.6098 26474460 PMC4741845

[8] Borah A Raveendran S Rochani A Maekawa T Kumar DS. Targeting self-renewal pathways in cancer stem cells: clinical implications for cancer therapy. Oncogenesis (2015) 4:e177. 10.1038/oncsis.2015.35 26619402 PMC4670961

[9] Peiris-Pagès M Martinez-Outschoorn UE Pestell RG Sotgia F Lisanti MP. Cancer stem cell metabolism. Breast Cancer Res (2016) 18(1):55. 10.1186/s13058-016-0712-6 27220421 PMC4879746

[10] Menendez JA. Metabolic control of cancer cell stemness: lessons from iPS cells. Cell Cycle (2015) 14(24):3801–11. 10.1080/15384101.2015.1022697 25738999 PMC4825763

[11] Currie E Schulze A Zechner R Walther TC Farese RV Jr. Cellular fatty acid metabolism and cancer. Cell Metab (2013) 18(2):153–61. 10.1016/j.cmet.2013.05.017 23791484 PMC3742569

[12] Sancho P Barneda D Heeschen C Hallmarks of cancer stem cell metabolism. Br J Cancer (2016) 114(12):1305–12. 10.1038/bjc.2016.152 27219018 PMC4984474

[13] Ito K Suda T. Metabolic requirements for the maintenance of self-renewing stem cells. Nat Rev Mol Cell Biol (2014) 15(4):243–56. 10.1038/nrm3772 24651542 PMC4095859

[14] Nakajima EC Van Houten B. Metabolic symbiosis in cancer: refocusing the Warburg lens. Mol Carcinog (2013) 52(5):329–37. 10.1002/mc.21863 22228080 PMC9972501

[15] Gao C Shen Y Jin F Miao Y Qiu X. Cancer stem cells in small cell lung cancer cell line H446: higher dependency on oxidative phosphorylation and mitochondrial substrate-level phosphorylation than non-stem cancer cells. PLoS One (2016) 11(5):e0154576. 10.1371/journal.pone.0154576 27167619 PMC4863974

[16] Liu PP Liao J Tang ZJ Wu WJ Yang J Zeng ZL Metabolic regulation of cancer cell side population by glucose through activation of the Akt pathway. Cell Death Differ (2014) 21(1):124–35. 10.1038/cdd.2013.131 24096870 PMC3857620

[17] Mamaeva V Niemi R Beck M Özliseli E Desai D Landor S Inhibiting notch activity in breast cancer stem cells by glucose functionalized nanoparticles carrying γ-secretase inhibitors. Mol Ther (2016) 24(5):926–36. 10.1038/mt.2016.42 26916284 PMC4881775

[18] Sansone P Ceccarelli C Berishaj M Chang Q Rajasekhar VK Perna F Self-renewal of CD133(hi) cells by IL6/Notch3 signalling regulates endocrine resistance in metastatic breast cancer. Nat Commun (2016) 7:10442. 10.1038/ncomms10442 26858125 PMC4748123

[19] Ciavardelli D Rossi C Barcaroli D Volpe S Consalvo A Zucchelli M Breast cancer stem cells rely on fermentative glycolysis and are sensitive to 2-deoxyglucose treatment. Cell Death Dis (2014) 5:e1336. 10.1038/cddis.2014.285 25032859 PMC4123079

[20] Isayev O Rausch V Bauer N Liu L Fan P Zhang Y Inhibition of glucose turnover by 3-bromopyruvate counteracts pancreatic cancer stem cell features and sensitizes cells to gemcitabine. Oncotarget (2014) 5(13):5177–89. 10.18632/oncotarget.2120 25015789 PMC4148131

[21] Shibuya K Okada M Suzuki S Seino M Seino S Takeda H Targeting the facilitative glucose transporter GLUT1 inhibits the self-renewal and tumor-initiating capacity of cancer stem cells. Oncotarget (2015) 6(2):651–61. 10.18632/oncotarget.2892 25528771 PMC4359246

[22] Li D Fu Z Chen R Zhao X Zhou Y Zeng B Inhibition of glutamine metabolism counteracts pancreatic cancer stem cell features and sensitizes cells to radiotherapy. Oncotarget (2015) 6(31):31151–63. 10.18632/oncotarget.5150 26439804 PMC4741594

[23] Sato M Kawana K Adachi K Fujimoto A Yoshida M Nakamura H Spheroid cancer stem cells display reprogrammed metabolism and obtain energy by actively running the tricarboxylic acid (TCA) cycle. Oncotarget (2016) 7(22):33297–305. 10.18632/oncotarget.8947 27120812 PMC5078095

[24] Sun P Xia S Lal B Shi X Yang KS Watkins PA Lipid metabolism enzyme ACSVL3 supports glioblastoma stem cell maintenance and tumorigenicity. BMC Cancer (2014) 14:401. 10.1186/1471-2407-14-401 24893952 PMC4055398

[25] Yasumoto Y Miyazaki H Vaidyan LK Kagawa Y Ebrahimi M Yamamoto Y Inhibition of fatty acid synthase decreases expression of stemness markers in glioma stem cells. PLoS One (2016) 11(1):e0147717. 10.1371/journal.pone.0147717 26808816 PMC4726602

[26] Ye H Adane B Khan N Sullivan T Minhajuddin M Gasparetto M Leukemic stem cells evade chemotherapy by metabolic adaptation to an adipose tissue niche. Cell Stem Cell (2016) 19(1):23–37. 10.1016/j.stem.2016.06.001 27374788 PMC4938766

[27] Saito Y Chapple RH Lin A Kitano A Nakada D. AMPK protects leukemia-initiating cells in myeloid leukemias from metabolic stress in the bone marrow. Cell Stem Cell (2015) 17(5):585–96. 10.1016/j.stem.2015.08.019 26440282 PMC4597792

[28] Barcellos-de-Souza P Gori V Bambi F Chiarugi P. Tumor microenvironment: bone marrow-mesenchymal stem cells as key players. Biochim Biophys Acta (2013) 1836:321–35. 10.1016/j.bbcan.2013.10.004 24183942

[29] Mishra PJ Humeniuk R Medina DJ Alexe G Mesirov JP Ganesan S Carcinoma-associated fibroblast-like differentiation of human mesenchymal stem cells. Cancer Res (2008) 68(11):4331–9. 10.1158/0008-5472.CAN-08-0943 18519693 PMC2725025

[30] Chiarugi P Cirri P Metabolic exchanges within tumor microenvironment. Cancer Lett (2016) 380:272–80. 10.1016/j.canlet.2015.10.027 26546872

[31] Fiaschi T Marini A Giannoni E Taddei ML Gandellini P De Donatis A Reciprocal metabolic reprogramming through lactate shuttle coordinately influences tumor-stroma interplay. Cancer Res (2012) 72:5130–40. 10.1158/0008-5472.CAN-12-1949 22850421

[32] Pavlides S Whitaker-Menezes D Castello-Cros R Flomenberg N Witkiewicz AK Frank PG The reverse Warburg effect: aerobic glycolysis in cancer associated fibroblasts and the tumor stroma. Cell Cycle (2009) 8(23):3984–4001. 10.4161/cc.8.23.10238 19923890

[33] Bonuccelli G Avnet S Grisendi G Salerno M Granchi D Dominici M Role of mesenchymal stem cells in osteosarcoma and metabolic reprogramming of tumor cells. Oncotarget (2014) 5(17):7575–88. 10.18632/oncotarget.2243 25277190 PMC4202145

[34] Martinez-Outschoorn UE Lin Z Whitaker-Menezes D Howell A Sotgia F Lisanti MP Ketone body utilization drives tumor growth and metastasis. Cell Cycle (2012) 11(21):3964–71. 10.4161/cc.22137 23082722 PMC3507492

[35] Martinez-Outschoorn UE Lin Z Whitaker-Menezes D Howell A Lisanti MP Sotgia F Ketone bodies and two-compartment tumor metabolism: stromal ketone production fuels mitochondrial biogenesis inepithelial cancer cells. Cell Cycle (2012) 11(21):3956–63. 10.4161/cc.22136 23082721 PMC3507491

[36] Pavlides S Tsirigos A Migneco G Whitaker-Menezes D Chiavarina B Flomenberg N The autophagic tumor stroma model of cancer: role of oxidative stress and ketone production in fueling tumor cell metabolism. Cell Cycle (2010) 9(17):3485–505. 10.4161/cc.9.17.12721 20861672 PMC3047615

[37] Wu D Zhuo L Wang X Metabolic reprogramming of carcinoma-associated fibroblasts and its impact on metabolic heterogeneity of tumors. Semin Cell Dev Biol (2016) 64:125–31. 10.1016/j.semcdb.2016.11.003 27833036

[38] Ko YH Lin Z Flomenberg N Pestell RG Howell A Sotgia F Glutamine fuels a vicious cycle of autophagy in the tumor stroma and oxidative mitochondrial metabolism in epithelial cancer cells: implications for preventing chemotherapy resistance. Cancer Biol Ther (2011) 12(12):1085–97. 10.4161/cbt.12.12.18671 22236876 PMC3335942

[39] Tang K Hu L Ma J Zhang H Zhang Y Li Y Brief report: human mesenchymal stem-like cells facilitate floating tumorigenic cell growth via glutamine-ammonium cycle. Stem Cells (2015) 33(9):2877–84. 10.1002/stem.2076 26031226

[40] Costa A Scholer-Dahirel A Mechta-Grigoriou F The role of reactive oxygen species and metabolism on cancer cells and their microenvironment. Semin Cancer Biol (2014) 25:23–32. 10.1016/j.semcancer.2013.12.007 24406211

[41] Martinez-Outschoorn UE Trimmer C Lin Z Whitaker-Menezes D Chiavarina B Zhou J Autophagy in cancer associated fibroblasts promotes tumor cell survival: role of hypoxia, HIF1 induction and NFκB activation in the tumor stromal microenvironment. Cell Cycle (2010) 9(17):3515–33. 10.4161/cc.9.17.12928 20855962 PMC3047617

[42] Caicedo A Fritz V Brondello JM Ayala M Dennemont I Abdellaoui N MitoCeption as a new tool to assess the effects of mesenchymal stem/stromal cell mitochondria on cancer cell metabolism and function. Sci Rep (2015) 5:9073. 10.1038/srep09073 25766410 PMC4358056

[43] Giatromanolaki A Koukourakis MI Koutsopoulos A Mendrinos S Sivridis E. The metabolic interactions between tumor cells and tumor-associated stroma (TAS) in prostatic cancer. Cancer Biol Ther (2012) 13:1284–9. 10.4161/cbt.21785 22895074 PMC3493436

[44] Rattigan YI Patel BB Ackerstaff E Sukenick G Koutcher JA Glod JW Lactate is a mediator of metabolic cooperation between stromal carcinoma associated fibroblasts and glycolytic tumor cells in the tumor microenvironment. Exp Cell Res (2012) 318:326–35. 10.1016/j.yexcr.2011.11.014 22178238 PMC3402174

[45] Peppicelli S Bianchini F Toti A Laurenzana A Fibbi G Calorini L. Extracellular acidity strengthens mesenchymal stem cells to promote melanoma progression. Cell Cycle (2015) 14(19):3088–100. 10.1080/15384101.2015.1078032 26496168 PMC4825622

[46] Zhao H Yang L Baddour J Achreja A Bernard V Moss T Tumor microenvironment derived exosomes pleiotropically modulate cancer cell metabolism. Elife (2016) 5:e10250. 10.7554/eLife.10250 26920219 PMC4841778

[47] Vallabhaneni KC Penfornis P Dhule S Guillonneau F Adams KV Mo YY Extracellular vesicles from bone marrow mesenchymal stem/stromal cells transport tumor regulatory microRNA, proteins, and metabolites. Oncotarget (2015) 6(7):4953–67. 10.18632/oncotarget.3211 25669974 PMC4467126

[48] Vallabhaneni KC Hassler MY Abraham A Whitt J Mo YY Atfi A Mesenchymal stem/stromal cells under stress increase osteosarcoma migration and apoptosis resistance via extracellular vesicle mediated communication. PLoS One (2016) 11(11):e0166027. 10.1371/journal.pone.0166027 27812189 PMC5094708

[49] Biswas SK Metabolic reprogramming of immune cells in cancer progression immunity. Immunity (2015) 43(3):435–49. 10.1016/j.immuni.2015.09.001 26377897

[50] Fejer G Sharma S Gyory I Self-renewing macrophages – a new line of enquiries in mononuclear phagocytes. Immunobiology (2015) 220(2):169–74. 10.1016/j.imbio.2014.11.005 25468723

[51] Guo Q Jin Z Yuan Y Liu R Xu T Wei H New mechanisms of tumor-associated macrophages on promoting tumor progression: recent research advances and potential targets for tumor immunotherapy. J Immunol Res (2016) 2016:9720912. 10.1155/2016/9720912 27975071 PMC5128713

[52] Xing Y Zhao S Zhou BP Mi J. Metabolic reprogramming of the tumour microenvironment. FEBS J (2015) 282(20):3892–8. 10.1111/febs.13402 26255648

[53] Roiniotis J Dinh H Masendycz P Turner A Elsegood CL Scholz GM Hypoxia prolongs monocyte/macrophage survival and enhanced glycolysis is associated with their maturation under aerobic conditions. J Immunol (2009) 182:7974–81. 10.4049/jimmunol.0804216 19494322

[54] Zhu L Zhao Q Yang T Ding W Zhao Y. Cellular metabolism and macrophage functional polarization. Int Rev Immunol (2015) 34:82–100. 10.3109/08830185.2014.969421 25340307

[55] He C Carter AB. The metabolic prospective and redox regulation of macrophage polarization. J Clin Cell Immunol (2015) 6(6):371. 10.4172/2155-9899.1000371 26962470 PMC4780841

[56] Rodríguez-Prados JC Través PG Cuenca J Rico D Aragonés J Martín-Sanz P Substrate fate in activated macrophages: a comparison between innate, classic, and alternative activation. J Immunol (2010) 185:605–14. 10.4049/jimmunol.0901698 20498354

[57] O’Neill LA Pearce EJ. Immunometabolism governs dendritic cell and macrophage function. J Exp Med (2016) 213(1):15–23. 10.1084/jem.20151570 26694970 PMC4710204

[58] Haschemi A Kosma P Gille L Evans CR Burant CF Starkl P The sedoheptulose kinase CARKL directs macrophage polarization through control of glucose metabolism. Cell Metab (2012) 15:813–26. 10.1016/j.cmet.2012.04.023 22682222 PMC3370649

[59] Jha AK Huang SC Sergushichev A Lampropoulou V Ivanova Y Loginicheva E Network integration of parallel metabolic and transcriptional data reveals metabolic modules that regulate macrophage polarization. Immunity (2015) 42:419–30. 10.1016/j.immuni.2015.02.005 25786174

[60] Tannahill GM Curtis AM Adamik J Palsson-McDermott EM McGettrick AF Goel G Succinate is an inflammatory signal that induces IL-1beta through HIF-1alpha. Nature (2013) 496:238–42. 10.1038/nature11986 23535595 PMC4031686

[61] Takeda N O’Dea EL Doedens A Kim JW Weidemann A Stockmann C Differential activation and antagonistic function of HIF-{alpha} isoforms in macrophages are essential for NO homeostasis. Genes Dev (2010) 24:491–501. 10.1101/gad.1881410 20194441 PMC2827844

[62] Covarrubias AJ Aksoylar HI Horng T. Control of macrophage metabolism and activation by mTOR and Akt signaling. Semin Immunol (2015) 27(4):286–96. 10.1016/j.smim.2015.08.001 26360589 PMC4682888

[63] Odegaard JI Chawla A Alternative macrophage activation and metabolism. Annu Rev Pathol (2011) 6:275–97. 10.1146/annurev-pathol-011110-130138 21034223 PMC3381938

[64] Mills EL O’Neill LA. Reprogramming mitochondrial metabolism in macrophages as an anti-inflammatory signal. Eur J Immunol (2016) 46(1):13–21. 10.1002/eji.201445427 26643360

[65] Braga TT Agudelo JS Camara NO. Macrophages during the fibrotic process: M2 as friend and foe. Front Immunol (2015) 6:602. 10.3389/fimmu.2015.00602 26635814 PMC4658431

[66] Huang SC Everts B Ivanova Y O’Sullivan D Nascimento M Smith AM Cell-intrinsic lysosomal lipolysis is essential for alternative activation of macrophages. Nat Immunol (2014) 15:846–55. 10.1038/ni.2956 25086775 PMC4139419

[67] Vats D Mukundan L Odegaard JI Zhang L Smith KL Morel CR Oxidative metabolism and PGC-1beta attenuate macrophage-mediated inflammation. Cell Metab (2006) 4:13–24. 10.1016/j.cmet.2006.08.006 16814729 PMC1904486

[68] Mukundan L Odegaard JI Morel CR Heredia JE Mwangi JW Ricardo-Gonzalez RR PPAR-delta senses and orchestrates clearance of apoptotic cells to promote tolerance. Nat Med (2009) 15:1266–72. 10.1038/nm.2048 19838202 PMC2783696

